# Edible Films Made of Dried Olive Leaf Extract and Chitosan: Characterization and Applications

**DOI:** 10.3390/foods11142078

**Published:** 2022-07-13

**Authors:** Michela Famiglietti, Alessandro Savastano, Rosa Gaglione, Angela Arciello, Daniele Naviglio, Loredana Mariniello

**Affiliations:** 1 Department of Chemical Sciences, University of Naples “Federico II”, 80126 Naples, Italy; michela.famiglietti@unina.it (M.F.); alessandro.savastano@icloud.com (A.S.); rosa.gaglione@unina.it (R.G.); angela.arciello@unina.it (A.A.); daniele.naviglio@unina.it (D.N.); 2 Center for Studies on Bioinspired Agro-Environmental Technology (BAT), University of Naples “Federico II”, 80126 Naples, Italy

**Keywords:** olive byproducts, chitosan, active packaging, food shelf life, antioxidants, circular economy

## Abstract

Nowadays a possible strategy in food preservation consists of the use of active and functional packaging to improve safety and ensure a longer shelf life of food products. Many studies refer to chitosan-based films because of the already-known chitosan (CH) antibacterial and antifungal activity. In this work, we developed CH-based films containing Dried Olive Leaf Extract (DOLE) obtained by Naviglio extractor, with the aim to investigate the polyphenols yield and the antioxidant activity of this extract entrapped in CH-based-edible films. Olive tree cultivation produces a huge amount of byproducts that are usually simply burned. Phenolic compounds are already studied for their beneficial effects on human health. Some studies reported that phenols isolated from olive leaves have been shown to inhibit the growth of different strains of microorganisms. Thus, the antimicrobial effect of DOLE-containing films against bacterial strains (*Salmonella enterica* subsp. *enterica serovar Typhimurium* ATCC^®^ 14028, *Salmonella enteritidis* RIVM 706, and *Enterococcus faecalis* ATCC^®^ 29212) was tested in vitro. The DOLE component of the films is effective in inhibiting all the bacteria tested in a dose-dependent manner. Thus, it was demonstrated that these edible films can act as active bioplastics when used to wrap hamburgers in substitution for baking paper, which is normally used.

## 1. Introduction

Chitosan (CH) is a natural linear copolymer derived from N-deacetylation of chitin and made up of units of N-acetyl-D-glucosamine and D-glucosamine (the latter exceeding 60%) [1], linked by beta-1,4 glycosidic linkage. Chitin is the second most abundant polymer on the earth, obtained from renewable sources, including exoskeletons of insects, arthropods such as shrimps, prawns, crabs, and cell walls of fungi [2].

Because of its biodegradability, biocompatibility, good film-forming capacity [3], and again because of its antibacterial and antifungal activity [4], CH is a very interesting polymer for applications in several industrial sectors.

The physical and chemical properties of CH depend on its molecular weight and degree of deacetylation. It presents one -NH_2_ group and two -OH groups on each repeating unit and shows a rigid crystalline structure due to inter and intra-molecular hydrogen bonding [2]. These groups have a fundamental role in some important physicochemical characteristics including solubility and mechanical properties [3].

CH is a weak base insoluble in water and organic solvents, but amino groups make it soluble under acidic conditions below pH 6.0 [5]. It was reported that CH is effectively soluble in several organic and inorganic acids such as acetic, citric, formic, lactic, and hydrochloric [6]. The pKa value of CH strictly depends on deacetylation degree, ionic strength and the charge neutralization of -NH_2_ groups [7], and the insoluble-soluble transition occurs usually between pH 6.5 and 6.0. Below this value, the amino groups get protonated, and CH becomes a soluble polymer [5].

The known antibacterial activity of CH can be influenced by different factors such as its average molecular weight [8] and the nature of organic acid [9] used for its dissolution. Besides the different structures of the cell wall of Gram-negative and Gram-positive bacteria, these are the reasons that can explain the diverse antimicrobial behavior of CH against both bacteria, as reported in some studies. Regardless, CH has been shown to act by different mechanisms: it can behave as a barrier that changes the cell permeability, preventing oxygen transference and thereby inhibiting the respiratory activity of bacteria [8]; it can absorb on the surface of the microbe by means of electrostatic interactions and create a thick polymeric membrane that prevents the entrance of necessary nutrients in the cells influencing physiological activities that are important for the growth of microbes [10].

Inside the cells, NH_3_^+^ groups of CH can interact with the negatively charged phosphoryl groups on the cell membrane, causing its deformation and distortion [11]; it can also diffuse until completely disrupting the cytoplasmatic membrane and provoking the leakage of electrolytes and the cell death [12]. Finally, CH can penetrate nuclei and bind DNA, inhibiting its replication ability, and can induce the synthesis of chitinase in fruit by increasing the gene expression of this enzyme that degrades the cell walls of microbes [13].

Since CH food safety (Generally Recognized as Safe (GRAS)) was improved by the US Food and Drug Administration in 2011 [14], we investigated the solubility of CH in citric acid (CA) to use this polymer as a matrix for delivering antioxidant compounds present in DOLE. Olive tree cultivation and the olive processing industry produce every year a huge amount of byproducts and residues [15]. Olive (*Olea europaea* L.) is one of the most cultivated ancient plants in the world, with about 9 million hectares occupied worldwide [16]. Typically, it grows in the tropical and temperate regions, and it represents a fundamental cultivar of the Mediterranean region, but it is also distributed in Western Asia, Arabian Peninsula, India, Northern Africa, and Iran [17]. The byproducts from the olive processing industry are mainly made up of leaves and branches obtained from the pruning of olive trees and harvesting and cleaning of olives [18]. The amount of leaves is about 25%, while branches represent around 75% of the dried weight from the total pruning residues, and they are generally used as animal feed or simply burned [19]. The chemical composition includes a lignocellulosic fraction, proteins, and extractives, with the latter reaching up to 45% [19]. Thus, various efforts toward better olive leaf waste management were carried out to extract energy or molecules. Among them, thermochemical treatments (combustion, gasification pyrolysis), biochemical treatments (anaerobic digestion and bioethanol production), drying methods (solar drying, microwave drying, freeze-drying), extraction methods, and condensation of active components are used to produce natural additives in different applicative fields [16]. Phenolic compounds, the main high-added value fraction of extractives present in olives leaves, are already studied for their several beneficial effects on human health. It was confirmed by different researchers the high antioxidant activity of these polyphenols and their effects as antihypertensive, cholesterol-lowering, cardioprotective, anti-inflammatory, antibacterial molecules and as coadjuvants in obesity treatment [20,21,22,23]. Origin, climatic conditions, moisture content, and proportions of the branches on the tree affect the chemical composition and the amount of polyphenolic compounds in olive leaves [16]. Polyphenols mainly present in olive leaves belong to five groups: oleuropeosides (oleuropein and verbascoside), flavones (luteolin-7-glucoside, apigenin-7-glucoside, diosmetin-7-glucoside, luteolin and diosmetin), flavonols (rutin), flavan-3-ols (catechin), and substituted phenols (tyrosol, hydroxytyrosol, vanillin, vanillic acid and caffeic acid) [24]. Among these the most abundant compound in olive leaves is oleuropein, which follows hydroxytyrosol, luteolin, apigenin, and verbascoside [25]. Hydroxytyrosol is more present in processed olive fruit and olive oil, while oleuropein is predominant in unprocessed olive fruit and leaves. This fact was explained due to the enzymatic reactions that occur during the maturation and processing of olives [24]. Oleuropein can prevent the formation of free radicals thanks to its ability to chelate metal ions such as Cu^2+^ and Fe^3+^ [26]. Moreover, both oleuropein and hydroxytyrosol have been shown to be scavengers of superoxide anions as well as of hypochlorous acid, a potent oxidant produced in vivo at the site of inflammation and a major component of chlorine-based bleaches that can often come into contact with food during manufacturing [27]. To date, different extraction methods were used to obtain high-added value from olive leaves, the traditional and most used is the solid-liquid extraction by maceration of the biomass in a solvent [28]. In the last years, new extraction methods are developed to reduce the amounts of solvent, time extraction, and sample preparation cost. Among them, supercritical fluid extraction and ultrasound-assisted extraction are more commonly used, followed by superheated liquid extraction, pressurized liquid extraction, and fractionation by solid-phase extraction. The selected extraction method, the nature of solvent, temperature, pH, and time of extraction are fundamental parameters that influence the yield and the content of polyphenolic compounds derived from olive leaves [29,30]. Obviously, the chemical characteristics of compounds make them less or more extractable by different methods: it was observed that compounds with less polarity as apigenin and luteolin are more easily obtainable by supercritical fluid extraction or pressurized liquid extraction, while maceration is more suitable to extract polar compounds such as oleuropein derivatives [31]. This work aimed to investigate the polyphenols yield of DOLE obtained by the Naviglio extractor and the antioxidant activity of this extract in chitosan-based-edible films. Our studies have also demonstrated the antioxidant activity of DOLE-containing films after in vitro oral digestion, which suggests the use of our films as a novel supplement for a diet rich in polyphenols. Moreover, we tested in vitro the antimicrobial effect of DOLE containing films against bacterial strains (*Salmonella typhimurium* ATCC^®^ 14028, *Salmonella enteriditis* RIVM 706, and *Enterococcus faecalis* ATCC^®^ 29212). In turn, the antimicrobial inhibitory effect of DOLE in meat samples over a time up to 20 days, in comparison to baking (parchment) paper normally used to store hamburgers, was demonstrated. We have also studied the mechanical properties of the films suggesting for DOLE a role as plasticizer. Thus, it can be assessed that multiple roles can be attributed to DOLE, nowadays a waste from olive trees pruning that can become a high-added-value product according to the principles of the circular economy.

## 2. Materials and Methods

### 2.1. Materials

CH (from shrimp shell, low molecular weight, Degree of Deacetylation ≥ 75%), CA (99%), urea, DPPH, NaCl were supplied by Sigma-Aldrich Company (St. Louis, MO, USA). Chemical reagents used for electrophoresis were from Bio-Rad (Segrate, Milano, Italy). Sodium hydroxide and hydrochloric acid were purchased from Sigma Aldrich Companyv (St.Louis, MO, USA). Glycerol (GLY), methanol and magnesium nitrate were from Carlo Erba S.p.A. (Milan, Italy) mTGase (Activa, WM, containing 1% of mTGase and 99% of maltodextrins) obtained from the culture of *Streptoverticillium* sp., was supplied by Prodotti Gianni (Milano, Italy). Folin–Ciocâlteu reagent was purchased by Fisher Bioreagents (Pittsburgh, PA, USA) Contact slide kit for controlling bacterial development (*Enterobacteriaceae* and *Streptococcus Faecalis*) in meat samples was purchased by Chimica Centro according to ISO 18593. Meat samples (hamburghers) were purchased from a local market. Olive leaf samples (cv “pisciottano”) were collected when the pruning is traditionally done from a farm in Cilento, a seaside land of the Campania Region, Southern Italy. Olive leaves were kept in sealed bags and stored at room temperature until use.

### 2.2. DOLE Preparation by Naviglio’s Principle

Before being submitted to the extraction process, olive leaves were dried in a static oven at 50 °C for two days. After that, 50 g of the samples were put in the filter bag (100 μm porosity), which was then inserted in the extraction chamber of the Naviglio extractor^®^ (Lab Model 500 cm^3^ capacity). Extraction was performed using 500 mL of H_2_O (containing 0.15% (*w*/*v*) CA, and 0.20% (*w*/*v*) potassium sorbate, pH 4.1) at room temperature and applying a 10-bar pressure for 2 min (static phase). After that, a remixing of liquid was applied for 2 min (dynamic phase). The pressure gradient is responsible for the extraction from the solid material (suction effect) as reported in Naviglio [32]. Liquid samples (10 mL), named DOLE, were collected after 2, 4, 6, and 24 h. DOLE samples were kept at 4 °C until they were used.

### 2.3. Total Polyphenols Content (TPC) Evaluation

DOLE TPC in samples obtained at different times of extraction, was determined by colorimetric in vitro assay using Folin–Ciocâlteau [33]. The calibration curve was set up with different concentrations of gallic acid (0.1–1 g/L). In particular, 0.1 mL of gallic acid solutions were mixed with 0.5 mL of 2 N Folin–Ciocâlteau reagent and 1.5 mL of freshly prepared 20% (*v*/*v*) Na_2_CO_3_, then the volume was adjusted to 10 mL with distilled water. Conversely, 0.1 mL of DOLE at different times of extraction (2-4-6-24 h) was mixed with the same reagents described. All the samples were incubated at room temperature in the dark for 1 h, then absorbance of the samples was measured at 765 nm using UV/visible Spectrophotometer (SmartSpec 3000 Bio-Rad, Segrate, Milan, Italy). The results were expressed as gallic acid equivalents (GAE). All determinations were carried out in triplicates.

### 2.4. Solubility of CH in CA

To investigate the solubility of CH in CA, different aqueous solutions of such acid were prepared (1.5–3.0% (*w*/*v*)). In each solution (50 mL) 1.5 g of CH were added under stirring. After 2 h, each solution was let stand for 1 h (without any stirring) to check the formation of precipitates. To quantify the CH dissolved, the supernatants were left to dry to obtain the dried matter. Solubility was expressed as percentage of the 1.5 g of CH added in each solution.

### 2.5. Preparation of Film Forming Solutions (FFSs) Containing DOLE

FFSs were prepared by using CA 3% (*w*/*v*) and chitosan 3% (*w*/*v*) at pH 2.5. DOLE was added in different volumes (10-15-20-40% (*v*/*v*)) after that its pH value was adjusted to 2.8 using CA 1 M. Finally, glycerol, used as a plasticizer, was mixed at 10% (*w*/*w*) (in respect to the weight of CH) in each type of FFS.

### 2.6. Zeta Potential and Particle Size Measurements

Zeta potential, average particle size, and polydispersity index of the FFSs were analyzed using the Zetasizer Nano-ZSP (Malvern^®^, Worcestershire, UK).

Three independent measurements were carried out on each sample of FFSs (1 mL) diluted to have a CH final concentration of 1 mg/mL.

Zetasizer Nano combines different techniques of Light Scattering to obtain a complete characterization of a colloidal system. Operating with a helium-neon laser at a fixed wavelength of 633 nm, it calculates the Zeta potential by using the Electrophoretic Light Scattering (ELS), the mean diameter of particles by using Dynamic Light Scattering (DLS) and the polydispersity index (PDI), which represents a value relative to the variance in the particle size distribution.

Three independent measurements were carried out on each sample of FFSs (1 mL) diluted to have a final concentration of 1 mg/mL.

### 2.7. Films Preparation

FFSs were poured in polypropylene Petri dishes (9 cm in diameter) and dried in a climatic chamber at 25 °C and 45% RH for 2 days. After that, the films were stored in a desiccator to balance the moisture content at 60% RH and room temperature during the subsequent analyses.

### 2.8. Antioxidant Activity

The activity to scavenge DPPH, 2,2-diphenyl-1-picrylhydrazyl, was measured for the solubilized films with all the different volumes of DOLE, for FFS 20% (*v*/*v*) and for DOLE solution (20% *v*/*v*) for one month, every 5 days.

Films were solubilized in CA 3% (*w*/*v*): 20 mg of each sample were dissolved in 500 μL of CA solution and subjected to sonication for 3 min.

For the *DPPH* assay, each sample was prepared adding to 900 μL of DPPH methanolic solution (0.005% (*w*/*v*)) 100 μL of different samples. The latter were incubated in dark for 30 min at room temperature. After that, absorbance was measured at 517 nm using a UV/visible Spectrophotometer (SmartSpec 3000 Bio-Rad, Segrate, Milan, Italy), considering methanol as blank and a sample with methanol added to DPPH solution as a control. The *DPPH scavenging activity* was calculated for each sample according to this equation:

%*DPPH scavenging activity* = (*A_control_* − *A_sample_*/*A_control_*) × 100.

### 2.9. In Vitro Oral Digestion

After one month, the film containing 20% (*v*/*v*) DOLE was subjected to in vitro oral digestion, i.e., under simulated oral conditions according to Giosafatto et al. [34] with some modifications, and then the DPPH scavenging activity of this solution was measured to study the release of polyphenols entrapped in chitosan films.

For this analysis, 20 mg of each type of film was incubated in 500 μL of Simulated Salivary Fluid (SSF, (150 mM of NaCl, 3 mM of urea, pH 6.9) for 7 min at 170 rpm, according to Giosafatto et al. [34].

### 2.10. Film Mechanical Properties and Thickness

An Instron universal testing instrument (model no. 5543A, Instron Engineering Corp., Norwood, MA, USA) was used for mechanical properties characterization. Tensile Strength (TS), Young’s Modulus (YM), and Elongation at Break (EB) were determined according to ASTM D882-18 (1997). Each film was cut into strips with a length of 40 mm and a width of 10 mm, and they were tested by using a 1 kN load cell and with a rate of grip separation of 20 mm/min. Film thickness was determined in five spots on each strip by using a digital micrometer (IP65 Alpa exacto, Alpa metrology Co., Pontoglio (BS), Italy, sensitivity 0.001 mm).

### 2.11. Bacterial Strains, Growth Conditions, and Antimicrobial Activity of DOLE-Based Films

Bacterial strains *Salmonella enteriditis* 706 RIVM, *Salmonella enterica* subsp. *Enterica serovar Typhimurium* (ATCC^®^ 14028) and *Enterococcus faecalis* ATCC^®^ 29212 were grown in Muller Hinton Broth (MHB, Becton Dickinson Difco, Franklin Lakes, NJ, USA) and on Tryptic Soy Agar (TSA; Oxoid Ltd., Hampshire, UK) as previously described [35]. In all the experiments, bacteria were inoculated and grown overnight in MHB at 37 °C.

In all the experiments to assess the antimicrobial activity of DOLE-based films, bacterial cells were inoculated and grown overnight in MHB at 37 °C. The next day, bacteria were transferred to a fresh Tryptic Soy Broth (TSB) tube and grown to mid- logarithmic phase. The antimicrobial activity of the films, containing or not containing DOLE, was tested by using a previously described experimental procedure [36]. Briefly, bacterial cells were diluted into TSB to approximately 2 × 10^7^ CFU/mL and inoculated into TSA plates. A 1.5-cm^2^ square of the edible film was placed into the center of the inoculated plate and pressed to ensure full contact with the agar surface. Plates were then incubated at 37 °C for 24 h and the bacterial growth underneath the film was evaluated.

### 2.12. DOLE Containing Film Capability to Contrast Bacterial Growth in Meat Samples

Hamburgers were prepared and both surfaces were covered with edible films containing DOLE at the following concentrations: 0%, 10%, 15%, and 20% (*v*/*v*). Hamburgers coated with baking paper were used as control. All samples were kept at 4 °C, but analyzed at different times (0 days, 5 days, 10 days, and 20 days) as follows: 10 g of each hamburger (from which the edible films or baking paper were removed) were homogenized in sterile conditions in the presence of 100 mL of H_2_O. Contact slides (designed for identifying *Enterobacteriaceae* and *Streptococcus Faecalis*) were dipped in these solutions for 30 s and used according to the manufacturer’s instructions of Liofilchem^®^-Contact slide 5 kit following ISO 18593.

### 2.13. Statistical Analysis

SPSS19 (Version 19, SPSS Inc., Chicago, IL, USA) software was used for all statistical analyses. One-way analysis of variance (ANOVA) and Duncan’s multiple range tests (*p* < 0.05) were used to determine the significant difference among the samples.

## 3. Results

### 3.1. Olive Leaf Extract Preparation and Total Polyphenol Content (TPC)

During pruning of olive trees, a huge amount of pruning wastes is produced in all countries where the olive tree cultivation is part of the local economy. It is well known that olive tree drupes can produce one of the best oils from a nutritional point of view. Olive oil is, in fact, rich with polyphenols and vitamin E, depending on the method used [36,37,38]. Also, the pruning waste is rich in valuable compounds, such as polyphenols. It is described that olive leaves are particularly plentiful in highly healthy molecules, such as hydroxytyrosol and oleuropein [24]. For this reason, many items can be found, especially in the online market, that are suggested as supplements and/or cosmetic purposes. In this paper we wanted to propose the use of polyphenols extracted by olive pruning wastes as antioxidants and antimicrobial components of CH-based films to be used as both supplements for an easy administration and as films to wrap hamburgers. To reach this aim we have obtained dried olive extracts (DOLE) by referring to the Naviglio methodology [32] Naviglio extractor is an innovative technology that allows to quickly extract, from solid materials, the extractable compounds in organic and inorganic solvents compared to conventional techniques. It is based on a suction effect theorized in “Naviglio’s Principle” [32]. Compression of liquid in which the solid matrix occurs at a pressure of around 8–10 bar by extracting solvent for a certain time is followed by an immediate decompression at atmospheric pressure. Thereby, a rapid release of extracting liquid, caused by a pressure gradient, is transported mechanically outside from the solid matrix the extractable compounds. Each extractive cycle consists of two phases: in the first one, also called the static phase, the system is under pressure and the liquid can penetrate the solid matrix; at the beginning of the second one, a negative pressure gradient is generated between the inside and the outside of the solid matrix to provoke the suction effect necessary to the extraction [32]. The extractions were performed at different times to assess the best protocol to obtain the highest quantity of polyphenolic molecules. As shown in Table 1, the TPC increases over time. Moreover, the amount of TPC results are notable, as expected from the literature [24]. It can be assessed that Naviglio’s method is very effective, as reported in Mirpoor et al. [39] In fact, these authors compared Naviglio’s method with maceration-based technology for the extraction of TPC from cardon leaves. Extraction in water was assessed to be more efficient than extraction with organic solvents, also by Sabry et al. [24]. Moreover, olive leaves derived from pruning contain, after 24 h of time extraction, a higher amount of TPC (6.6 ± 0.1 g GAE/L) compared to cardon (0.15 ± 0.1 g GAE/L), suggesting the importance of recovering olive leaves wastes. It is worth to note that TPC is equal to 4.4 ± 0.2 g GAE/L after only 2 h of time extraction.

### 3.2. Chitosan Solubility in Citric Acid

**Figure 1:**
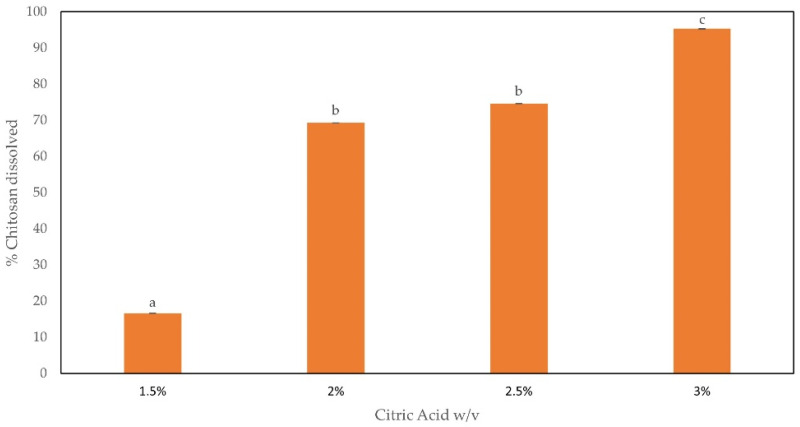
CH solubility, expressed in percentage, when dissolved in CA. Different small letters (a–c) indicate significant differences among the values reported in each bar (*p* < 0.05).

Because of amino groups, CH is soluble in acidic solutions among which there is CA that is food grade. However, the solubilization depends on the degree of deacetylation (DD). Thus, we have performed experiments to assess the CA concentration to achieve the maximum solubility of CH. CA was used in substitution of acetic acid normally used in our previous papers, where chitosan-based bioplastics were prepared to be proposed to wrap foods [40]. In this work we decided to recur to CA since our main aim was to use chitosan-base edible films to convey phenolic compounds present in DOLE as supplements with a pleasant taste for consumers (which is not insured using acetic acid as the solvent). The maximum solubility was achieved using 3% (*w*/*v*) CA, as shown in Figure 1. Thus, further analyses were performed using 3% (*w*/*v*) CH dissolved in 3% (*w*/*v*) CA.

### 3.3. FFSs Characterization and Preparation of Films by Casting

**Figure 2:**
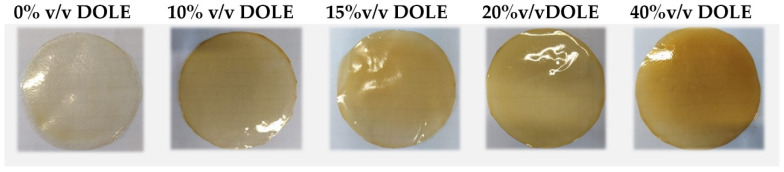
Chitosan-based films containing different volumes of DOLE (0–40% (*v*/*v*)).

FFSs prepared in absence and in presence of increasing concentrations of DOLE were analyzed via Zeta sizer to determine the Zeta potential, particle size, and PDI. Table 2 shows that all the solutions were highly stable. The addition of DOLE provoked a lowering in the Zeta potential value in respect to the control (CH-based solution in absence of DOLE), which turned out higher by increasing the amount of DOLE. The mean particle size of DOLE containing samples were lower than the one exhibited by the control and decreased by increasing the DOLE concentration. The same observation can be done regarding PDI values. The decreasing in mean particle size could be due to aggregation of CH particles provoked by polyphenolic molecules present in DOLE. These solutions were used to cast films. Obtained films are shown in Figure 2. Films appear transparent but yellowish depending on the DOLE content.

### 3.4. Antioxidant Activity (AA) of DOLE-Containing Films over the Time

**Figure 3:**
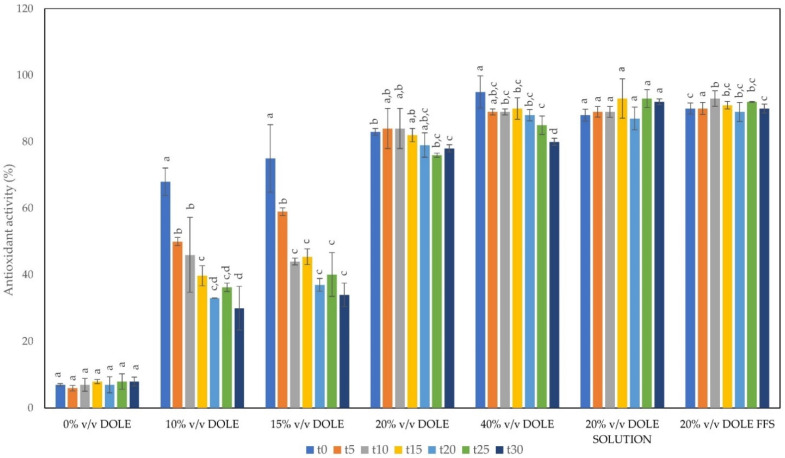
Antioxidant activity by DPPH assay of solubilized films (DOLE 0-10-15-20-40% (*v*/*v*)), 20% (*v*/*v*) DOLE solutions, and 20% (*v*/*v*) DOLE Film Forming Solutions (FFS) at different days of storage. Different small letters (a–d) indicate significant differences among the values reported in each bar (*p* < 0.05).

Figure 3 shows the AA of solubilized DOLE containing films detected from 0 time and every 5 days up to 30. Lower AA was observed in films containing 10 and 15% (*v*/*v*) DOLE even after few days of storage. Instead, the AA remained high in both kinds of films containing 20% and 40% (*v*/*v*) DOLE, respectively. The AA was also detected over the time in 20% (*v*/*v*) DOLE solutions and in 20% (*v*/*v*) DOLE FFS. It can be noticed that the AA values in solubilized films were lower than DOLE-containing solutions and FFS. In Figure 3, we report only the results obtained with 20% (*v*/*v*) DOLE containing samples, but similar results were obtained with 40% (*v*/*v*) DOLE samples. A possible explanation could be that the phenolic compounds present in DOLE exert a lower activity when entrapped in the chitosan film matrix. A reduction of AA due to cardoon leaf extracts (CLE) entrapped in cardoon protein-based films was also reported in Mirpoor et al. [39], even though for a longer time (70 days). It must be noticed that DOLE-containing films showed a high AA even after 30 days (70%) compared to CLE-containing films at time 0 (60%) [39].

### 3.5. In Vitro Oral Digestion

**Figure 4:**
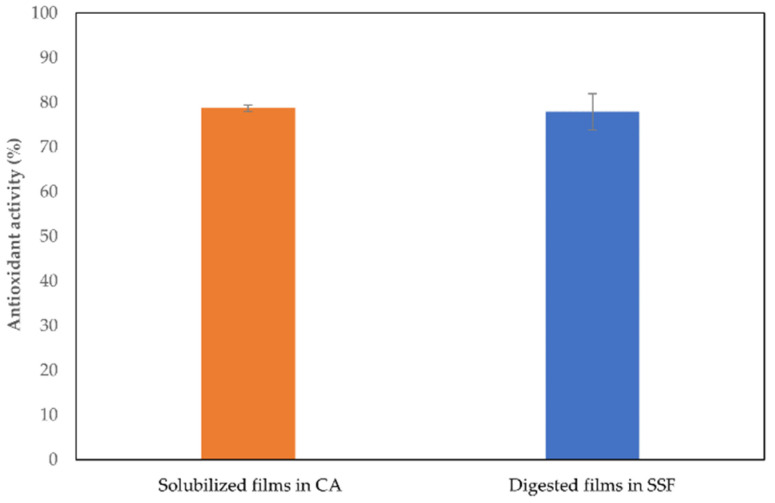
Antioxidant activity by DPPH assay of 20% (*v*/*v*) DOLE films subjected to in vitro oral digestion after 30 days compared to the AA of solubilized films after 30 days.

In order to ensure the release of polyphenols entrapped in CH films, samples (20% *v/v* DOLE) stored for 30 days were subjected to in vitro oral digestion, under simulated oral conditions, and then the DPPH scavenging activity of these solutions was measured. The results in Figure 4 showed that the release of polyphenols from 30 days storage films occurred properly after in vitro oral digestion. The AA results of such films were compared to the ones obtained from the controls represented by solubilized films stored for the same time (30 days). The AA of both groups of different samples are perfectly comparable, demonstrating that polyphenols entrapped in the films are still active after in vitro simulated oral digestion. Thus, we can affirm that DOLE containing films could be used to delivery polyphenols in a novel supplement for an effective application into the nutraceutical industry.

### 3.6. Mechanical Properties

Since DOLE visually seemed to have a plasticizer effect on chitosan-based films, we studied the mechanical properties of these films to verify the possible influence of DOLE on Tensile Strength (TS), Elongation at Break (EB), and Young’s Modulus (YM). Samples thickness was also measured.

**Figure 5:**
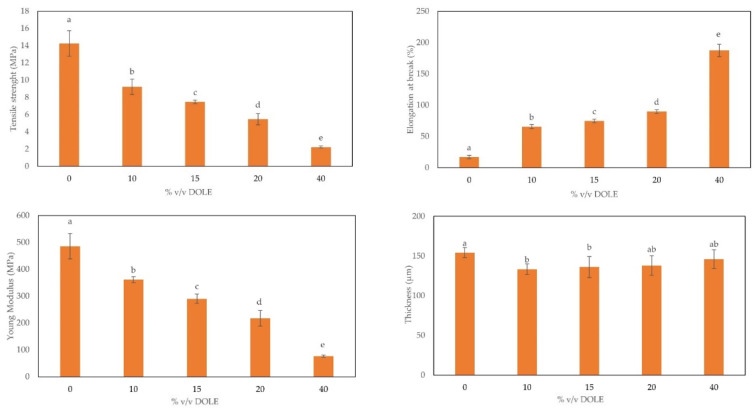
Properties of chitosan-based films with different % (*v*/*v*) of DOLE (0–40%). On the top: from left to right Tensile Strength and Elongation at Break. On the bottom: from left to right Young’s Modulus and Thickness. Different small letters (a–e) indicate significant differences among the values reported in each bar (*p* < 0.05).

As shown in Figure 5, the medium thickness of all samples does not differ significatively from the control. Regarding mechanical properties, we note that they are influenced by the presence of DOLE. We can observe, proportionally with the increment of DOLE concentration, an increase of EB, and a decrease of TS and YM, also with respect to the control without DOLE. These results suggest a role as plasticizer for DOLE in addition to the one exerted by glycerol. Similar results were obtained by Mariniello et al., using grape juice as a plasticizer in *N. sativa* protein-based films [41].

### 3.7. Antimicrobial Activity of Films Containing DOLE

**Figure 6:**
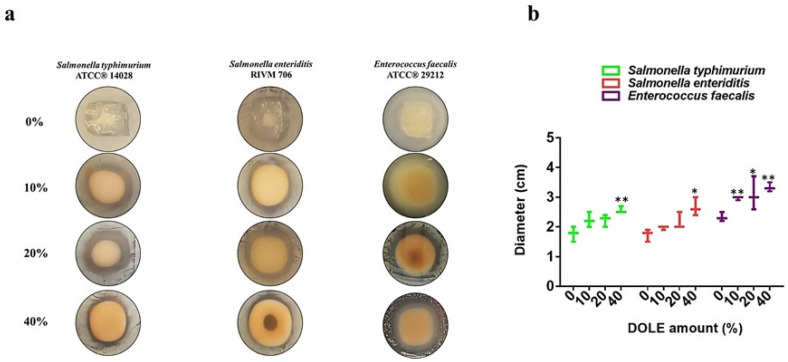
Antimicrobial activity of DOLE-based films. Antimicrobial effects were evaluated by analyzing the growth of the bacterial cells held in direct contact with the films (**a**). The diameter of the inhibition circle was determined for each bacterial strain in contact with the different films and shown in the graph (**b**). The experiments were carried out in triplicate. Statistical analyses were performed by using Student’s *t*-test. Significant differences were indicated as * *p* < 0.05 or ** *p* < 0.01.

**Figure 7:**
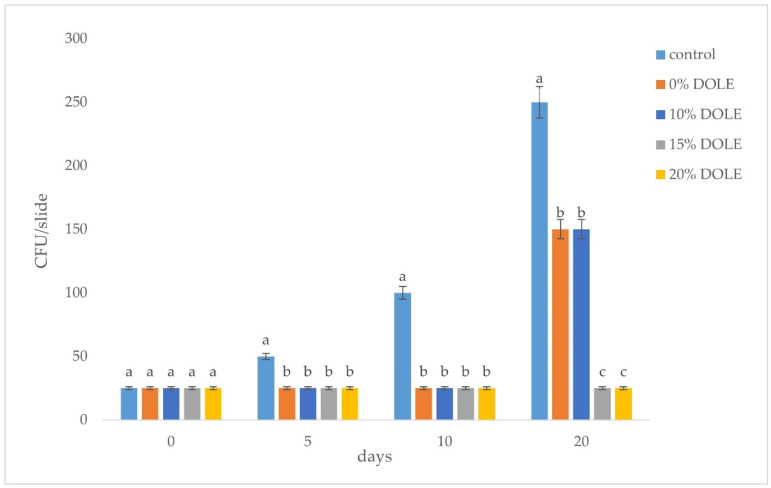
CFU (*Enterobacteriaceae*) in meat samples covered with DOLE-containing films. Controls represent samples covered with baking paper. Different small letters (a–c) indicate significant differences among the values reported in each bar (*p* < 0.05).

Films prepared in the absence or in the presence of DOLE at different concentrations (10%, 20%, or 40% (*v*/*v*)) were analyzed for their antimicrobial activity towards Gram-negative bacteria (*S. enteriditis* 706 RIVM, *S. enterica* subsp. *enterica serovar* Typhimurium ATCC^®^ 14028) and a Gram-positive bacterial strain *E. faecalis* ATCC^®^ 29212. Usually, these strains are involved in food contamination and spoilage. In particular, the presence of *E. faecalis* suggests that a contamination by the two other pathogen strains may occur. Thus, small squares (1.5 cm × 1.5 cm) of each film were placed into the inoculated plate in full contact with the agar surface. After 24 h at 37 °C, only the films containing DOLE were found to be able to significantly inhibit bacterial growth with the strongest effects observed at the highest content of DOLE (Figure 6a,b). The DOLE component of the films is effective in inhibiting the growth of all the bacteria tested in a dose dependent manner. Other studies suggested a broad antimicrobial activity of olive leaves extract in a concentration dependent-manner [42]. Taking advantage of these results, we have tested the capability of DOLE-containing films as active bioplastics, able to control the bacterial spoilage of meat hamburgers over time. To this aim, we have used the Liofilchem^®^ contact slide 5 kit, which allows selective detection of *Enterobacteriaceae* and enterococci. In particular, employed slides contain media to selectively identify *E. coli* ATCC^®^ 25922 and *Enterococcus faecalis* ATCC^®^ 19433. As samples, we have chosen meat hamburgers and covered each side with our edible films containing increasing amounts of DOLE. As controls we used hamburgers coated with baking paper, as is commonly done. We kept all samples stored at 4 °C for different times (0, 5, 10, and 20 days). Afterwards, meat samples were treated as described in materials and method in Section 2.12. As shown in Figure 7, the most effective edible films are the ones containing 15% and 20% (*v*/*v*) DOLE after 20 days storage, demonstrating that the antimicrobial activity of phenolic compounds is still valuable over time, especially against *E. coli*. Samples were also tested for the presence of *E. faecalis* bacteria, that were totally absent even in the control at time 0 (data not shown). Thus, we can assess that DOLE-containing films exhibit antimicrobial activity against all the bacteria tested both in vitro (Figure 6) and as edible films used to protect hamburgers over time (Figure 7).

## 4. Conclusions

In the present paper it was demonstrated that olive byproducts can be recovered to obtain a high added-value product possibly used in the food industry. It showed the effectiveness of polyphenols contained in DOLE and entrapped in a chitosan-based matrix in acting as antimicrobials and antioxidants. Obtained films can be classified as edible and, thus, could be used as supplements to reinforce the human diet with polyphenols. Moreover, such films can be used to wrap meat hamburgers to delay spoilage during storage due to common microbial contaminants.

## Figures and Tables

**Table 1:** Total phenolic content in DOLE at different times of extraction.

Hours	Polyphenols (g GAE/L)
2	4.4 ± 0.2 ^a^
4	4.6 ± 0.1 ^a^
6	5.5 ± 0.2 ^b^
24	6.6 ± 0.4 ^c^

Different small letters (a–c) indicate significant differences among the values reported in each column (*p* < 0.05).

**Table 2:** Zeta potential, particle size, and PDI of FFSs containing DOLE (10–40% (*v*/*v*)). The control was set up in the absence of DOLE.

DOLE in FFSs % (*v*/*v*)	Z Potential (mV)	Mean Particle Size (nm)	PDI
0	55.71 ± 4.61 ^a^	567.00 ± 19.33 ^a^	0.67 ± 0.02 ^a^
10	40.08 ± 3.20 ^c^	568.96 ± 31.40 ^a^	0.48 ± 0.01 ^b^
15	43.59 ± 2.51 ^c^	500.68 ± 28.30 ^b^	0.50 ± 0.03 ^b^
20	49.37 ± 5.42 ^b^	426.22 ± 49.05 ^c^	0.51 ± 0.05 ^b^
40	53.55 ± 5.61 ^a,b^	419.32 ± 73.02 ^c^	0.51 ± 0.05 ^b^

Different small letters (a–c) indicate significant differences among the values reported in each column (*p* < 0.05).

## Data Availability

The data presented in this study are available on request from the corresponding author.
